# [5,10,15,20-Tetra­kis­(penta­fluoro­phen­yl)porphyrin­ato]zinc(II) benzene disolvate

**DOI:** 10.1107/S2414314620008779

**Published:** 2020-07-10

**Authors:** Zeyuan Lin, Jianfeng Li

**Affiliations:** a College of Materials Science and Opto-electronic Technology, CAS Center for Excellence in Topological Quantum Computation & Center of Materials Science and Optoelectronics Engineering, University of Chinese Academy of Sciences, Yanqi Lake, Huairou District, Beijing 101408, People’s Republic of China; University Koblenz-Landau, Germany

**Keywords:** crystal structure, zinc porphyrin, hydrogen inter­action, π–π inter­action

## Abstract

Single crystals of the title zinc porphyrinato complex, [Zn(C_44_H_8_F_20_N_4_)]·2C_6_H_6_, were obtained by the solvent evaporation method. The molecular complex exhibits point group symmetry  with the central Zn^II^ atom located on an inversion centre. The porphyrinato core is approximately planar, and the cation has no other ligating atoms than the four porphyrinato N atoms. π–π inter­actions between benzene solvent mol­ecules and [Zn(TFPP)] units lead to multilayer packing structures. In addition, inter­molecular C—H⋯F hydrogen bonding is observed between [Zn(TFPP)] mol­ecules.

## Abstract 2

The porphyrinato core of the title zinc porphyrinato complex is approximately planar, and the cation has no other ligating atoms than the four porphyrinato N atoms. The molecular complex exhibits point group symmetry 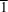 with the central Zn^II^ atom located on an inversion centre.

## Graphical abstract

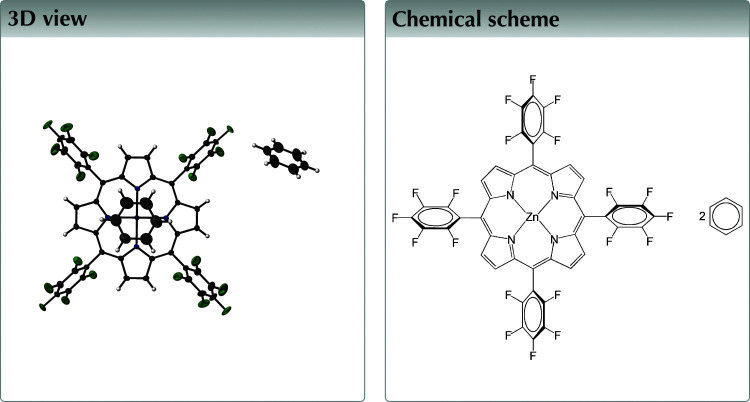

## Structure description

Metalloporphyrin derivatives have been investigated extensively, not only as heme models but also as functional building blocks of mol­ecular materials and devices. Zinc porphyrins have been employed as photosensitizers (Liu *et al.*, 2006 ▸) and chemical sensors (Wu *et al.*, 2016 ▸) because of their good photoelectronic and chemical stabilities. In 1995, Gray and co-workers published the first zinc porphyrin crystal structure, (5,10,15,20-tetra­kis­(penta­fluoro­phen­yl)porphyrinato)zinc(II) *n*-hexane solvate, which crystallizes in the space group *P*2_1_/c and shows a planar conformation of the porphyrin ligand (Birnbaum *et al.*, 1995 ▸). In this work, a new zinc penta­fluoro­phenyl-porphyrin crystal structure, [Zn(TFPP)]·2C_6_H_6_ (TFPP is the tetrakis(penta­fluor­phenyl)porphyrinato ligand), is reported.

**Figure 1:**
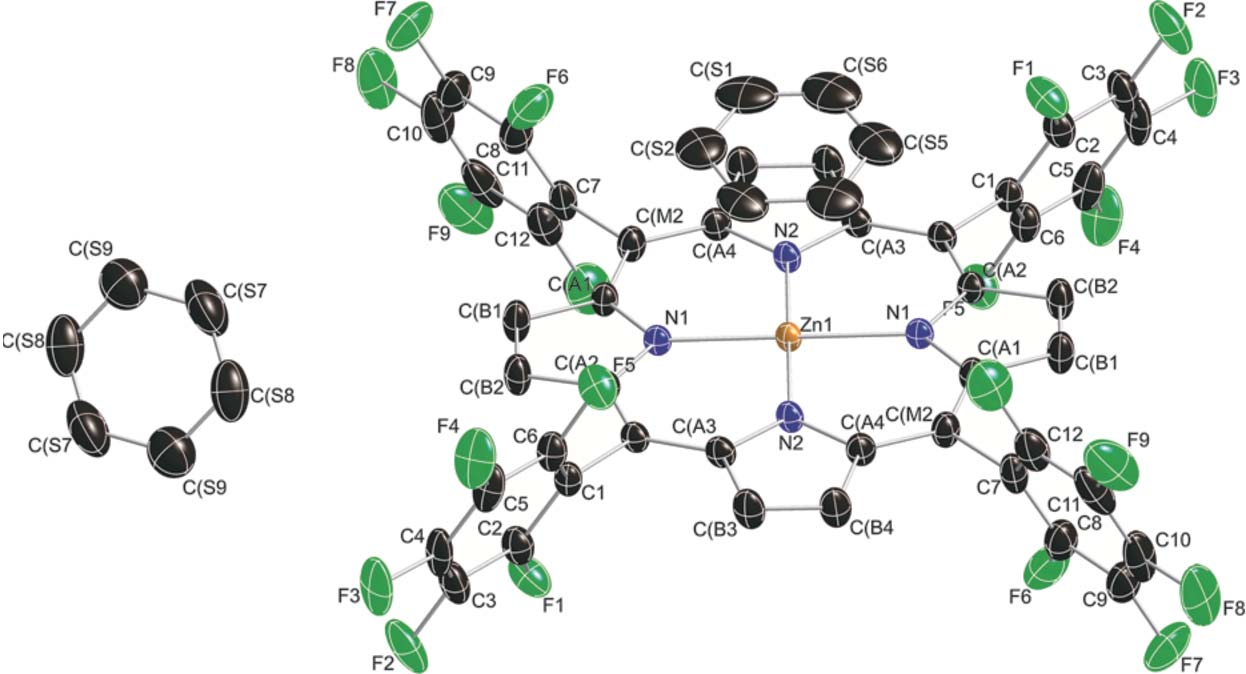
*ORTEP* diagram of the mol­ecular structure of the title compound with displacement ellipsoids drawn at the 50% probability level. Only one orientation of the disordered solvent benzene mol­ecule (CS1–CS6) is shown. H atoms are omitted for clarity.

**Figure 2:**
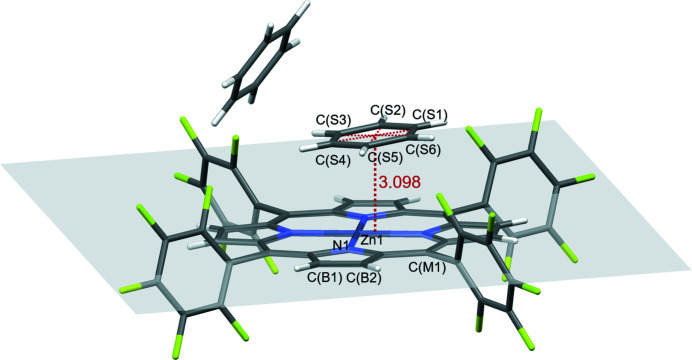
Schematic illustration of π–π stacking in the crystal structure of the title compound showing the distance between the centroid of benzene ring (*X*cent) and the porphyrinato plane.

The mol­ecular entities of the title compound are shown in Fig. 1 ▸. The structural features of the current complex are similar to those of the previously reported [Zn(TFPP)] *n*-hexane solvate (Birnbaum *et al.*, 1995 ▸). As can be seen, the porphyrinato core is approximately planar (r.m.s. deviation = 0.03 Å). Two benzene solvent mol­ecules are found besides the [Zn(TFPP)] units. One of them is found to be disordered (CS1–CS6) over two positions. π–π stacking inter­actions are observed between the disordered benzene mol­ecules and [Zn(TFPP)] moieties, as illustrated in Fig. 2 ▸. The distance between the centroid of the benzene ring (*X*_cent_) and the porphyrinato plane is 3.098 Å, which is consistent with the reported values for related complexes (about 3.33–3.45 Å; Uno *et al.*, 2003 ▸).

**Figure 3:**
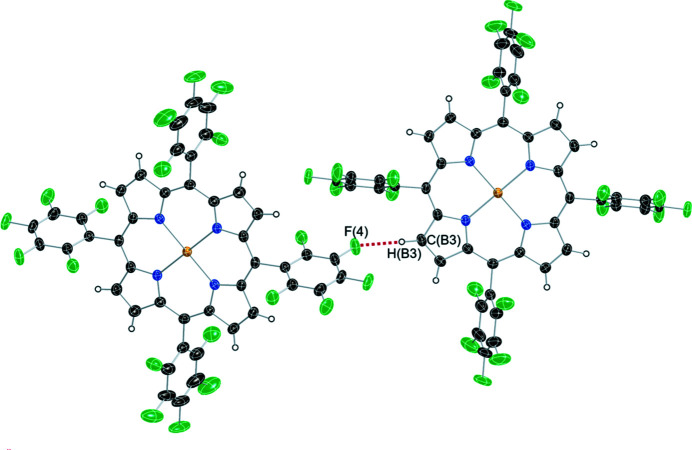
C—H⋯F hydrogen bonds in the crystal structure of the title compound.

**Figure 4:**
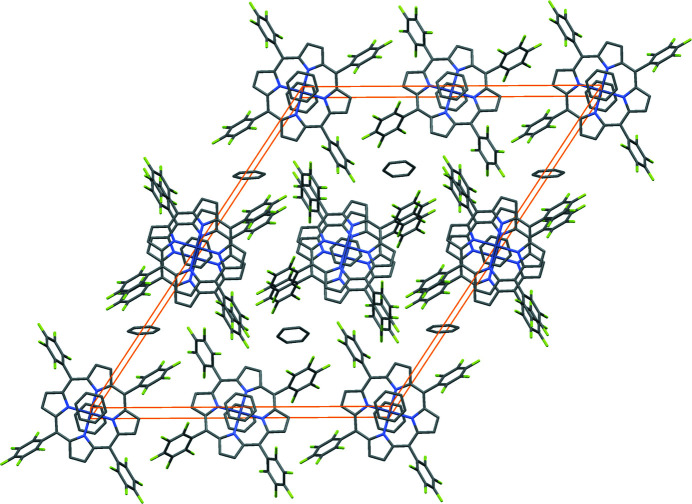
A view of the packing pattern of the title compound with the unit cell indicated by orange lines. H atoms are omitted for clarity.

Inter­molecular non-bonding inter­actions are also found for the title compound. As given in Fig. 3 ▸ and Table 1 ▸, the distance between CB3 and F4, HB3 and F4 are 3.276 and 2.36 Å, respectively, being consistent with C–H⋯F hydrogen bonds (3.455 and 2.53 Å, respectively; Thamotharan *et al.*, 2003 ▸). The packing pattern of the title compound is shown in Fig. 4 ▸.

## Synthesis and crystallization

**General Information.** All operations were accomplished by standard Schlenk techniques unless otherwise specified. Benzene, which was used for crystallization of the final products, was washed with concentrated sulfuric acid and then with sodium bicarbonate solution at least three times, dried with anhydrous magnesium sulfate and distilled with calcium hydride, sodium and di­phenyl­ketone, sequentially under argon atmosphere. The precursor compound H_2_(TFPP) was synthesized by the method reported by Lindsey *et al.* (1987 ▸).

**Synthesis of [5,10,15,20-tetra­kis­(penta­fluoro­phen­yl)porphyrinato]zinc(II) benzene disolvate**

H_2_(TFPP) (500 mg, 0.51 mmol) and Zn(Ac)_2_·2H_2_O (2.25 g, 10.26 mmol) were dissolved in 250 ml of CH_3_OH and 250 ml of CHCl_3_. After the solution had been transferred to a conical flask (1000 ml), it was refluxed under stirring for 4 h under argon. The raw products were washed with deionized water to remove the remaining zinc salts. The organic phases were dried and purified by column chromatography using silica and eluting the product with hexa­ne/di­chloro­methane (2:1). Red–pink [Zn(TFPP)] powder (yield 489 mg, 0.47 mmol, 91.8%), was obtained after solvent evaporation. Red single crystals of the title compound were grown by slow solvent evaporation of a solution of the title compound in freshly distilled benzene at room temperature.

## Refinement

Crystal data, data collection and structure refinement details are summarized in Table 2 ▸. A solvent benzene mol­ecule is observed to be disordered over two positions of equal occupancy (CS1–CS6). These atoms and corresponding bonds were restrained by ISOR and RIGU commands, respectively.

## Supplementary Material

Crystal structure: contains datablock(s) I, New_Global_Publ_Block. DOI: 10.1107/S2414314620008779/im4008sup1.cif

Supporting information file. DOI: 10.1107/S2414314620008779/im4008Isup3.cdx

Structure factors: contains datablock(s) I. DOI: 10.1107/S2414314620008779/im4008Isup4.hkl

CCDC reference: 2013055

Additional supporting information:  crystallographic information; 3D view; checkCIF report

## Figures and Tables

**Table 1:** Hydrogen-bond geometry (Å, °)

*D*—H⋯*A*	*D*—H	H⋯*A*	*D*⋯*A*	*D*—H⋯*A*
C*B*3—H*B*3⋯F4^i^	0.95	2.36	3.276 (2)	163

Symmetry code: (i) 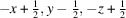 .

**Table 2:** Experimental details

Crystal data	
Chemical formula	[Zn(C_44_H_8_F_20_N_4_)]·2C_6_H_6_
*M*_r_	1194.13
Crystal system, space group	Monoclinic, *C*2/*c*
Temperature (K)	100
*a*, *b*, *c* (Å)	34.176 (7), 6.3699 (13), 26.382 (5)
β (°)	123.428 (6)
*V* (Å^3^)	4793.4 (17)
*Z*	4
Radiation type	Mo *K*α
μ (mm^−1^)	0.64
Crystal size (mm)	0.42 × 0.14 × 0.12

Data collection	
Diffractometer	Bruker D8 QUEST System
Absorption correction	Multi-scan (*SADABS*; Bruker, 2014 ▸)
*T*_min_, *T*_max_	0.656, 0.746
No. of measured, independent and observed [*I* > 2σ(*I*)] reflections	22042, 5287, 4369
*R*_int_	0.044
(sin θ/λ)_max_ (Å^−1^)	0.645

Refinement	
*R*[*F* ^2^ > 2σ(*F* ^2^)], *wR*(*F* ^2^), *S*	0.034, 0.119, 0.91
No. of reflections	5287
No. of parameters	367
H-atom treatment	H-atom parameters constrained
Δρ_max_, Δρ_min_ (e Å^−3^)	0.38, −0.38

Computer programs: *APEX2* and *SAINT* (Bruker, 2014 ▸), *SHELXT2014/6* (Sheldrick, 2015*a* ▸), *SHELXL2014/6* (Sheldrick, 2015*b* ▸), *OLEX2* (Dolomanov *et al.*, 2009 ▸), *Mercury* (Macrae *et al.*, 2020 ▸) and *enCIFer* (Allen *et al.*, 2004 ▸).

## full crystallographic data

### Crystal data

**Table:**

[Zn(C_44_H_8_F_20_N_4_)]·2C_6_H_6_	*F*(000) = 2376
*M**_r_* = 1194.13	*D*_x_ = 1.655 Mg m^−^^3^
Monoclinic, *C*2/*c*	Mo *K*α radiation, λ = 0.71073 Å
*a* = 34.176 (7) Å	Cell parameters from 9674 reflections
*b* = 6.3699 (13) Å	θ = 2.9–27.1°
*c* = 26.382 (5) Å	µ = 0.64 mm^−^^1^
β = 123.428 (6)°	*T* = 100 K
*V* = 4793.4 (17) Å^3^	Block, red
*Z* = 4	0.42 × 0.14 × 0.12 mm

### Data collection

**Table:**

Bruker D8 QUEST System diffractometer	4369 reflections with *I* > 2σ(*I*)
Radiation source: fine-focus sealed tube	*R*_int_ = 0.044
f\ and ω scans	θ_max_ = 27.3°, θ_min_ = 2.4°
Absorption correction: multi-scan (SADABS; Bruker, 2014)	*h* = −42→43
*T*_min_ = 0.656, *T*_max_ = 0.746	*k* = −8→8
22042 measured reflections	*l* = −33→33
5287 independent reflections	

### Refinement

**Table:**

Refinement on *F*^2^	0 restraints
Least-squares matrix: full	Hydrogen site location: inferred from neighbouring sites
*R*[*F*^2^ > 2σ(*F*^2^)] = 0.034	H-atom parameters constrained
*wR*(*F*^2^) = 0.119	*w* = 1/[σ^2^(*F*_o_^2^) + (0.1*P*)^2^] where *P* = (*F*_o_^2^ + 2*F*_c_^2^)/3
*S* = 0.91	(Δ/σ)_max_ < 0.001
5287 reflections	Δρ_max_ = 0.38 e Å^−^^3^
367 parameters	Δρ_min_ = −0.38 e Å^−^^3^

### Special details

**Table:**

Geometry. All esds (except the esd in the dihedral angle between two l.s. planes) are estimated using the full covariance matrix. The cell esds are taken into account individually in the estimation of esds in distances, angles and torsion angles; correlations between esds in cell parameters are only used when they are defined by crystal symmetry. An approximate (isotropic) treatment of cell esds is used for estimating esds involving l.s. planes.
Refinement. All of hydrogen atoms were placed in calculated positions (C–H = 0.95 Å for aryl hydrogen atoms) and were refined using a riding model with fixed isotropic displacement parameters of *U*_iso_(H) = 1.2*U*_eq_(C).

### Fractional atomic coordinates and isotropic or equivalent isotropic displacement parameters (Å^2^)

**Table:**

	*x*	*y*	*z*	*U*_iso_*/*U*_eq_	Occ. (<1)
Zn1	0.000000	0.500000	0.000000	0.01507 (8)	
F1	0.12626 (4)	0.03956 (18)	0.20870 (5)	0.0382 (3)	
F2	0.20705 (5)	−0.0107 (2)	0.31796 (6)	0.0552 (4)	
F3	0.27217 (4)	0.2974 (2)	0.36575 (5)	0.0529 (4)	
F4	0.25736 (4)	0.6539 (2)	0.30139 (5)	0.0505 (4)	
F5	0.17700 (4)	0.70796 (18)	0.19275 (5)	0.0360 (3)	
F6	0.04641 (5)	0.0847 (2)	−0.14163 (6)	0.0527 (3)	
F7	0.09198 (7)	0.0230 (3)	−0.19701 (7)	0.0770 (5)	
F8	0.15178 (5)	0.3220 (3)	−0.18992 (7)	0.0782 (6)	
F9	0.16562 (5)	0.6763 (3)	−0.12718 (7)	0.0732 (5)	
F10	0.12069 (5)	0.7385 (2)	−0.07077 (6)	0.0499 (3)	
N1	0.01956 (5)	0.4839 (2)	0.08851 (6)	0.0164 (3)	
N2	0.06745 (5)	0.4424 (2)	0.02711 (6)	0.0171 (3)	
C1	0.14878 (6)	0.3755 (3)	0.19688 (7)	0.0203 (3)	
C2	0.15802 (6)	0.1941 (3)	0.23036 (8)	0.0268 (4)	
C3	0.19933 (7)	0.1660 (3)	0.28668 (8)	0.0341 (5)	
C4	0.23254 (6)	0.3214 (4)	0.31069 (7)	0.0341 (5)	
C5	0.22491 (6)	0.5017 (3)	0.27841 (8)	0.0323 (4)	
C6	0.18339 (6)	0.5273 (3)	0.22238 (8)	0.0250 (4)	
C7	0.08204 (6)	0.4135 (3)	−0.10438 (8)	0.0280 (4)	
C8	0.07564 (7)	0.2338 (4)	−0.13712 (9)	0.0373 (5)	
C9	0.09912 (9)	0.2002 (4)	−0.16577 (10)	0.0505 (7)	
C10	0.12929 (8)	0.3496 (5)	−0.16194 (10)	0.0525 (7)	
C11	0.13627 (8)	0.5295 (4)	−0.13032 (10)	0.0488 (7)	
C12	0.11267 (7)	0.5597 (4)	−0.10180 (9)	0.0365 (5)	
C(A1	−0.00926 (6)	0.5061 (3)	0.10911 (7)	0.0196 (3)	
C(A2	0.06336 (6)	0.4409 (3)	0.13817 (7)	0.0179 (3)	
C(A3	0.10493 (6)	0.4068 (3)	0.08512 (7)	0.0194 (3)	
C(A4	0.08383 (6)	0.4208 (3)	−0.00964 (7)	0.0212 (3)	
C(B1	0.01740 (6)	0.4734 (3)	0.17401 (7)	0.0279 (4)	
H(B1	0.005686	0.478869	0.199334	0.034*	
C(B2	0.06203 (6)	0.4336 (3)	0.19194 (7)	0.0259 (4)	
H(B2	0.087686	0.406087	0.232232	0.031*	
C(B3	0.14611 (6)	0.3610 (3)	0.08474 (8)	0.0290 (4)	
H(B3	0.176594	0.330989	0.119056	0.035*	
C(B4	0.13301 (6)	0.3690 (3)	0.02628 (8)	0.0305 (4)	
H(B4	0.152511	0.345089	0.011582	0.037*	
C(M1	0.10343 (5)	0.4091 (3)	0.13702 (7)	0.0178 (3)	
C(M2	0.05699 (6)	0.4455 (3)	−0.07278 (7)	0.0219 (4)	
C(S1	0.0408 (4)	0.950 (2)	−0.0123 (6)	0.061 (2)	0.5
H(S1	0.063403	0.919040	−0.021508	0.073*	0.5
C(S2	−0.0044 (4)	1.008 (2)	−0.0588 (6)	0.051 (2)	0.5
H(S2	−0.012183	1.013446	−0.099368	0.061*	0.5
C(S3	−0.0380 (3)	1.0566 (19)	−0.0472 (5)	0.044 (2)	0.5
H(S3	−0.068634	1.098603	−0.078945	0.053*	0.5
C(S7	0.23680 (11)	0.9084 (5)	0.45930 (12)	0.0655 (8)	
H(S7	0.227437	1.020489	0.431141	0.079*	
C(S8	0.25387 (8)	0.7307 (5)	0.45095 (10)	0.0526 (6)	
H(S8	0.256539	0.717365	0.417107	0.063*	
C(S9	0.26728 (10)	0.5702 (5)	0.49140 (12)	0.0621 (7)	
H(S9	0.279376	0.444178	0.485916	0.075*	
C(S4	−0.0252 (4)	1.042 (2)	0.0146 (6)	0.056 (2)	0.5
H(S4	−0.047227	1.073430	0.024683	0.067*	0.5
C(S5	0.0196 (4)	0.981 (2)	0.0585 (6)	0.055 (3)	0.5
H(S5	0.028285	0.968400	0.099303	0.066*	0.5
C(S6	0.0526 (4)	0.938 (2)	0.0447 (6)	0.059 (3)	0.5
H(S6	0.083646	0.900416	0.076063	0.071*	0.5

### Atomic displacement parameters (Å^2^)

**Table:**

	*U*^11^	*U*^22^	*U*^33^	*U*^12^	*U*^13^	*U*^23^
Zn1	0.01178 (13)	0.01950 (14)	0.01282 (12)	0.00114 (10)	0.00607 (10)	0.00089 (10)
F1	0.0328 (6)	0.0353 (7)	0.0349 (6)	−0.0018 (5)	0.0113 (5)	0.0102 (5)
F2	0.0484 (8)	0.0659 (9)	0.0353 (7)	0.0174 (7)	0.0130 (6)	0.0308 (6)
F3	0.0218 (6)	0.1041 (11)	0.0158 (5)	0.0120 (7)	−0.0003 (5)	0.0052 (6)
F4	0.0253 (6)	0.0807 (10)	0.0319 (6)	−0.0229 (6)	0.0070 (5)	−0.0161 (6)
F5	0.0313 (6)	0.0373 (6)	0.0319 (6)	−0.0102 (5)	0.0126 (5)	0.0000 (5)
F6	0.0606 (9)	0.0553 (8)	0.0539 (8)	−0.0003 (7)	0.0390 (7)	−0.0154 (7)
F7	0.0994 (14)	0.0922 (13)	0.0626 (10)	0.0365 (10)	0.0592 (10)	−0.0045 (8)
F8	0.0732 (11)	0.1355 (15)	0.0649 (9)	0.0625 (10)	0.0627 (9)	0.0491 (10)
F9	0.0530 (9)	0.1079 (13)	0.0862 (11)	0.0131 (9)	0.0558 (9)	0.0405 (10)
F10	0.0426 (8)	0.0614 (9)	0.0549 (8)	−0.0066 (6)	0.0326 (7)	0.0057 (7)
N1	0.0126 (6)	0.0205 (7)	0.0149 (6)	0.0002 (5)	0.0068 (5)	0.0004 (5)
N2	0.0126 (7)	0.0224 (7)	0.0141 (6)	0.0013 (5)	0.0061 (5)	0.0019 (5)
C1	0.0142 (8)	0.0306 (9)	0.0158 (7)	0.0029 (7)	0.0080 (6)	0.0012 (6)
C2	0.0200 (9)	0.0366 (11)	0.0218 (8)	0.0025 (8)	0.0103 (7)	0.0040 (7)
C3	0.0278 (10)	0.0502 (12)	0.0220 (9)	0.0134 (9)	0.0122 (8)	0.0140 (8)
C4	0.0142 (9)	0.0689 (15)	0.0126 (8)	0.0074 (9)	0.0032 (7)	0.0016 (8)
C5	0.0152 (8)	0.0577 (13)	0.0211 (8)	−0.0088 (9)	0.0083 (7)	−0.0105 (9)
C6	0.0178 (8)	0.0373 (11)	0.0191 (8)	−0.0011 (7)	0.0095 (7)	−0.0007 (7)
C7	0.0191 (9)	0.0490 (11)	0.0184 (8)	0.0120 (8)	0.0118 (7)	0.0094 (8)
C8	0.0342 (11)	0.0547 (13)	0.0280 (10)	0.0143 (10)	0.0203 (9)	0.0055 (9)
C9	0.0555 (15)	0.0727 (17)	0.0333 (11)	0.0347 (13)	0.0308 (11)	0.0130 (11)
C10	0.0469 (14)	0.091 (2)	0.0399 (12)	0.0413 (14)	0.0370 (12)	0.0326 (13)
C11	0.0287 (11)	0.0841 (19)	0.0445 (12)	0.0188 (11)	0.0272 (10)	0.0344 (13)
C12	0.0265 (10)	0.0562 (14)	0.0299 (10)	0.0113 (9)	0.0175 (9)	0.0131 (9)
C(A1	0.0176 (8)	0.0261 (9)	0.0158 (7)	0.0002 (7)	0.0096 (6)	0.0002 (6)
C(A2	0.0152 (8)	0.0211 (8)	0.0146 (7)	−0.0011 (6)	0.0064 (6)	−0.0009 (6)
C(A3	0.0146 (8)	0.0232 (9)	0.0174 (7)	0.0020 (7)	0.0070 (6)	0.0022 (6)
C(A4	0.0170 (8)	0.0288 (9)	0.0192 (8)	0.0039 (7)	0.0108 (7)	0.0027 (7)
C(B1	0.0203 (9)	0.0476 (12)	0.0167 (8)	0.0051 (8)	0.0106 (7)	0.0024 (7)
C(B2	0.0188 (9)	0.0414 (10)	0.0144 (8)	0.0024 (8)	0.0072 (7)	0.0009 (7)
C(B3	0.0157 (9)	0.0482 (12)	0.0207 (8)	0.0095 (8)	0.0084 (7)	0.0069 (8)
C(B4	0.0193 (9)	0.0510 (12)	0.0230 (9)	0.0111 (8)	0.0130 (8)	0.0067 (8)
C(M1	0.0140 (8)	0.0208 (8)	0.0146 (7)	0.0006 (6)	0.0053 (6)	0.0007 (6)
C(M2	0.0179 (8)	0.0319 (10)	0.0185 (8)	0.0029 (7)	0.0117 (7)	0.0021 (7)
C(S1	0.067 (5)	0.021 (3)	0.084 (4)	−0.005 (4)	0.035 (4)	−0.009 (3)
C(S2	0.065 (5)	0.022 (3)	0.058 (4)	−0.010 (4)	0.030 (4)	−0.009 (3)
C(S3	0.048 (5)	0.019 (3)	0.056 (3)	−0.010 (4)	0.022 (3)	0.001 (2)
C(S7	0.075 (2)	0.0692 (19)	0.0419 (14)	−0.0035 (16)	0.0254 (14)	0.0182 (13)
C(S8	0.0390 (13)	0.082 (2)	0.0325 (11)	−0.0139 (13)	0.0172 (10)	0.0024 (12)
C(S9	0.0566 (17)	0.0699 (18)	0.0503 (15)	0.0061 (14)	0.0235 (13)	0.0037 (13)
C(S4	0.065 (5)	0.026 (4)	0.072 (4)	−0.014 (4)	0.035 (4)	−0.007 (3)
C(S5	0.066 (6)	0.023 (4)	0.059 (4)	−0.009 (5)	0.024 (4)	0.000 (3)
C(S6	0.062 (5)	0.024 (3)	0.071 (4)	−0.010 (4)	0.023 (4)	−0.006 (3)

### Geometric parameters (Å, º)

**Table:**

Zn1—N1	2.0470 (13)	C(A1—C(B1	1.444 (2)
Zn1—N1^i^	2.0469 (13)	C(A1—C(M2^i^	1.397 (2)
Zn1—N2^i^	2.0355 (14)	C(A2—C(B2	1.445 (2)
Zn1—N2	2.0354 (14)	C(A2—C(M1	1.401 (2)
F1—C2	1.338 (2)	C(A3—C(B3	1.443 (2)
F2—C3	1.333 (2)	C(A3—C(M1	1.398 (2)
F3—C4	1.342 (2)	C(A4—C(B4	1.441 (2)
F4—C5	1.340 (2)	C(A4—C(M2	1.399 (2)
F5—C6	1.340 (2)	C(B1—H(B1	0.9500
F6—C8	1.336 (3)	C(B1—C(B2	1.349 (3)
F7—C9	1.338 (3)	C(B2—H(B2	0.9500
F8—C10	1.338 (2)	C(B3—H(B3	0.9500
F9—C11	1.340 (3)	C(B3—C(B4	1.350 (2)
F10—C12	1.340 (3)	C(B4—H(B4	0.9500
N1—C(A1	1.370 (2)	C(S1—H(S1	0.9500
N1—C(A2	1.368 (2)	C(S1—C(S2	1.390 (13)
N2—C(A3	1.369 (2)	C(S1—C(S6	1.327 (16)
N2—C(A4	1.368 (2)	C(S2—H(S2	0.9500
C1—C2	1.381 (2)	C(S2—C(S3	1.378 (11)
C1—C6	1.382 (2)	C(S3—H(S3	0.9500
C1—C(M1	1.498 (2)	C(S3—C(S4	1.438 (15)
C2—C3	1.386 (2)	C(S7—H(S7	0.9500
C3—C4	1.370 (3)	C(S7—C(S8	1.345 (4)
C4—C5	1.367 (3)	C(S7—C(S9^ii^	1.389 (4)
C5—C6	1.383 (2)	C(S8—H(S8	0.9500
C7—C8	1.376 (3)	C(S8—C(S9	1.362 (4)
C7—C12	1.375 (3)	C(S9—H(S9	0.9500
C7—C(M2	1.502 (2)	C(S4—H(S4	0.9500
C8—C9	1.389 (3)	C(S4—C(S5	1.371 (14)
C9—C10	1.366 (4)	C(S5—H(S5	0.9500
C10—C11	1.359 (4)	C(S5—C(S6	1.390 (12)
C11—C12	1.386 (3)	C(S6—H(S6	0.9500
			
N1^i^—Zn1—N1	180.0	C(M1—C(A2—C(B2	125.19 (15)
N2^i^—Zn1—N1^i^	89.73 (5)	N2—C(A3—C(B3	109.96 (14)
N2^i^—Zn1—N1	90.27 (5)	N2—C(A3—C(M1	125.19 (15)
N2—Zn1—N1	89.73 (5)	C(M1—C(A3—C(B3	124.83 (15)
N2—Zn1—N1^i^	90.27 (5)	N2—C(A4—C(B4	109.97 (14)
N2—Zn1—N2^i^	180.0	N2—C(A4—C(M2	125.22 (15)
C(A1—N1—Zn1	126.40 (10)	C(M2—C(A4—C(B4	124.81 (15)
C(A2—N1—Zn1	126.86 (10)	C(A1—C(B1—H(B1	126.4
C(A2—N1—C(A1	106.70 (12)	C(B2—C(B1—C(A1	107.21 (15)
C(A3—N2—Zn1	127.14 (11)	C(B2—C(B1—H(B1	126.4
C(A4—N2—Zn1	126.56 (11)	C(A2—C(B2—H(B2	126.6
C(A4—N2—C(A3	106.22 (13)	C(B1—C(B2—C(A2	106.90 (14)
C2—C1—C6	116.16 (15)	C(B1—C(B2—H(B2	126.6
C2—C1—C(M1	122.19 (15)	C(A3—C(B3—H(B3	126.6
C6—C1—C(M1	121.64 (15)	C(B4—C(B3—C(A3	106.83 (15)
F1—C2—C1	119.77 (15)	C(B4—C(B3—H(B3	126.6
F1—C2—C3	118.04 (16)	C(A4—C(B4—H(B4	126.5
C1—C2—C3	122.19 (17)	C(B3—C(B4—C(A4	107.01 (15)
F2—C3—C2	120.67 (19)	C(B3—C(B4—H(B4	126.5
F2—C3—C4	119.50 (17)	C(A2—C(M1—C1	116.98 (14)
C4—C3—C2	119.82 (18)	C(A3—C(M1—C1	117.13 (14)
F3—C4—C3	120.24 (19)	C(A3—C(M1—C(A2	125.90 (14)
F3—C4—C5	120.15 (19)	C(A1^i^—C(M2—C7	117.14 (14)
C5—C4—C3	119.61 (16)	C(A1^i^—C(M2—C(A4	126.41 (15)
F4—C5—C4	119.98 (17)	C(A4—C(M2—C7	116.44 (15)
F4—C5—C6	120.29 (18)	C(S2—C(S1—H(S1	119.6
C4—C5—C6	119.72 (17)	C(S6—C(S1—H(S1	119.6
F5—C6—C1	119.74 (15)	C(S6—C(S1—C(S2	120.8 (10)
F5—C6—C5	117.77 (16)	C(S1—C(S2—H(S2	119.3
C1—C6—C5	122.49 (17)	C(S3—C(S2—C(S1	121.3 (10)
C8—C7—C(M2	121.29 (18)	C(S3—C(S2—H(S2	119.3
C12—C7—C8	116.60 (17)	C(S2—C(S3—H(S3	121.1
C12—C7—C(M2	122.11 (18)	C(S2—C(S3—C(S4	117.7 (9)
F6—C8—C7	119.98 (16)	C(S4—C(S3—H(S3	121.1
F6—C8—C9	118.0 (2)	C(S8—C(S7—H(S7	119.6
C7—C8—C9	122.0 (2)	C(S8—C(S7—C(S9^ii^	120.8 (3)
F7—C9—C8	120.1 (2)	C(S9^ii^—C(S7—H(S7	119.6
F7—C9—C10	120.5 (2)	C(S7—C(S8—H(S8	120.3
C10—C9—C8	119.4 (2)	C(S7—C(S8—C(S9	119.5 (2)
F8—C10—C9	120.5 (3)	C(S9—C(S8—H(S8	120.3
F8—C10—C11	119.3 (3)	C(S7^ii^—C(S9—H(S9	120.1
C11—C10—C9	120.21 (19)	C(S8—C(S9—C(S7^ii^	119.7 (3)
F9—C11—C10	119.9 (2)	C(S8—C(S9—H(S9	120.1
F9—C11—C12	120.6 (3)	C(S3—C(S4—H(S4	120.7
C10—C11—C12	119.5 (2)	C(S5—C(S4—C(S3	118.6 (8)
F10—C12—C7	120.01 (17)	C(S5—C(S4—H(S4	120.7
F10—C12—C11	117.7 (2)	C(S4—C(S5—H(S5	119.3
C7—C12—C11	122.3 (2)	C(S4—C(S5—C(S6	121.5 (11)
N1—C(A1—C(B1	109.49 (14)	C(S6—C(S5—H(S5	119.3
N1—C(A1—C(M2^i^	125.00 (14)	C(S1—C(S6—C(S5	120.1 (11)
C(M2^i^—C(A1—C(B1	125.51 (15)	C(S1—C(S6—H(S6	119.9
N1—C(A2—C(B2	109.69 (14)	C(S5—C(S6—H(S6	119.9
N1—C(A2—C(M1	125.11 (14)		
			
Zn1—N1—C(A1—C(B1	177.00 (11)	C7—C8—C9—C10	−0.5 (3)
Zn1—N1—C(A1—C(M2^i^	−3.4 (2)	C8—C7—C12—F10	−179.99 (17)
Zn1—N1—C(A2—C(B2	−177.07 (11)	C8—C7—C12—C11	−0.2 (3)
Zn1—N1—C(A2—C(M1	3.0 (2)	C8—C7—C(M2—C(A1^i^	−74.3 (2)
Zn1—N2—C(A3—C(B3	176.82 (12)	C8—C7—C(M2—C(A4	104.8 (2)
Zn1—N2—C(A3—C(M1	−1.7 (2)	C8—C9—C10—F8	−179.00 (18)
Zn1—N2—C(A4—C(B4	−176.65 (12)	C8—C9—C10—C11	0.0 (3)
Zn1—N2—C(A4—C(M2	3.5 (3)	C9—C10—C11—F9	−179.4 (2)
F1—C2—C3—F2	0.3 (3)	C9—C10—C11—C12	0.3 (3)
F1—C2—C3—C4	−179.38 (17)	C10—C11—C12—F10	179.60 (19)
F2—C3—C4—F3	−1.2 (3)	C10—C11—C12—C7	−0.2 (3)
F2—C3—C4—C5	179.30 (17)	C12—C7—C8—F6	179.98 (18)
F3—C4—C5—F4	0.9 (3)	C12—C7—C8—C9	0.5 (3)
F3—C4—C5—C6	−178.28 (16)	C12—C7—C(M2—C(A1^i^	106.3 (2)
F4—C5—C6—F5	−0.4 (3)	C12—C7—C(M2—C(A4	−74.6 (2)
F4—C5—C6—C1	−179.69 (16)	C(A1—N1—C(A2—C(B2	0.59 (18)
F6—C8—C9—F7	0.4 (3)	C(A1—N1—C(A2—C(M1	−179.32 (16)
F6—C8—C9—C10	−179.91 (19)	C(A1—C(B1—C(B2—C(A2	−0.1 (2)
F7—C9—C10—F8	0.7 (3)	C(A2—N1—C(A1—C(B1	−0.67 (18)
F7—C9—C10—C11	179.8 (2)	C(A2—N1—C(A1—C(M2^i^	178.91 (17)
F8—C10—C11—F9	−0.4 (3)	C(A3—N2—C(A4—C(B4	0.3 (2)
F8—C10—C11—C12	179.32 (18)	C(A3—N2—C(A4—C(M2	−179.58 (17)
F9—C11—C12—F10	−0.7 (3)	C(A3—C(B3—C(B4—C(A4	0.3 (2)
F9—C11—C12—C7	179.52 (18)	C(A4—N2—C(A3—C(B3	−0.10 (19)
N1—C(A1—C(B1—C(B2	0.5 (2)	C(A4—N2—C(A3—C(M1	−178.62 (17)
N1—C(A2—C(B2—C(B1	−0.3 (2)	C(B2—C(A2—C(M1—C1	−3.2 (3)
N1—C(A2—C(M1—C1	176.69 (15)	C(B2—C(A2—C(M1—C(A3	177.12 (17)
N1—C(A2—C(M1—C(A3	−3.0 (3)	C(B3—C(A3—C(M1—C1	4.3 (3)
N2—C(A3—C(B3—C(B4	−0.1 (2)	C(B3—C(A3—C(M1—C(A2	−176.03 (17)
N2—C(A3—C(M1—C1	−177.39 (16)	C(B4—C(A4—C(M2—C7	0.5 (3)
N2—C(A3—C(M1—C(A2	2.3 (3)	C(B4—C(A4—C(M2—C(A1^i^	179.43 (18)
N2—C(A4—C(B4—C(B3	−0.4 (2)	C(M1—C1—C2—F1	1.5 (2)
N2—C(A4—C(M2—C7	−179.70 (17)	C(M1—C1—C2—C3	−177.79 (16)
N2—C(A4—C(M2—C(A1^i^	−0.7 (3)	C(M1—C1—C6—F5	−1.2 (2)
C1—C2—C3—F2	179.67 (17)	C(M1—C1—C6—C5	178.05 (16)
C1—C2—C3—C4	−0.1 (3)	C(M1—C(A2—C(B2—C(B1	179.63 (17)
C2—C1—C6—F5	−179.83 (15)	C(M1—C(A3—C(B3—C(B4	178.40 (18)
C2—C1—C6—C5	−0.5 (3)	C(M2—C7—C8—F6	0.5 (3)
C2—C1—C(M1—C(A2	70.4 (2)	C(M2—C7—C8—C9	−178.91 (18)
C2—C1—C(M1—C(A3	−109.85 (19)	C(M2—C7—C12—F10	−0.5 (3)
C2—C3—C4—F3	178.53 (16)	C(M2—C7—C12—C11	179.22 (17)
C2—C3—C4—C5	−1.0 (3)	C(M2^i^—C(A1—C(B1—C(B2	−179.08 (18)
C3—C4—C5—F4	−179.54 (17)	C(M2—C(A4—C(B4—C(B3	179.49 (19)
C3—C4—C5—C6	1.2 (3)	C(S1—C(S2—C(S3—C(S4	−1.3 (19)
C4—C5—C6—F5	178.84 (16)	C(S2—C(S1—C(S6—C(S5	1 (2)
C4—C5—C6—C1	−0.5 (3)	C(S2—C(S3—C(S4—C(S5	0.3 (17)
C6—C1—C2—F1	−179.89 (15)	C(S3—C(S4—C(S5—C(S6	1.2 (19)
C6—C1—C2—C3	0.8 (3)	C(S7—C(S8—C(S9—C(S7^ii^	0.0 (5)
C6—C1—C(M1—C(A2	−108.05 (19)	C(S9^ii^—C(S7—C(S8—C(S9	0.0 (5)
C6—C1—C(M1—C(A3	71.7 (2)	C(S4—C(S5—C(S6—C(S1	−2 (2)
C7—C8—C9—F7	179.81 (18)	C(S6—C(S1—C(S2—C(S3	1 (2)

Symmetry codes: (i) −*x*, −*y*+1, −*z*; (ii) −*x*+1/2, −*y*+3/2, −*z*+1.

### Hydrogen-bond geometry (Å, º)

**Table:**

*D*—H···*A*	*D*—H	H···*A*	*D*···*A*	*D*—H···*A*
C(*B*3—H(*B*3···F4^iii^	0.95	2.36	3.276 (2)	163

Symmetry code: (iii) −*x*+1/2, *y*−1/2, −*z*+1/2.
